# Cd^2+^ Toxicity to a Green Alga *Chlamydomonas reinhardtii* as Influenced by Its Adsorption on TiO_2_ Engineered Nanoparticles

**DOI:** 10.1371/journal.pone.0032300

**Published:** 2012-03-05

**Authors:** Wei-Wan Yang, Ai-Jun Miao, Liu-Yan Yang

**Affiliations:** State Key Laboratory of Pollution Control and Resource Reuse, School of the Environment, Nanjing University, Nanjing, Jiangsu Province, China; Massey University, New Zealand

## Abstract

In the present study, Cd^2+^ adsorption on polyacrylate-coated TiO_2_ engineered nanoparticles (TiO_2_-ENs) and its effect on the bioavailability as well as toxicity of Cd^2+^ to a green alga *Chlamydomonas reinhardtii* were investigated. TiO_2_-ENs could be well dispersed in the experimental medium and their pH_pzc_ is approximately 2. There was a quick adsorption of Cd^2+^ on TiO_2_-ENs and a steady state was reached within 30 min. A pseudo-first order kinetics was found for the time-related changes in the amount of Cd^2+^ complexed with TiO_2_-ENs. At equilibrium, Cd^2+^ adsorption followed the Langmuir isotherm with the maximum binding capacity 31.9, 177.1, and 242.2 mg/g when the TiO_2_-EN concentration was 1, 10, and 100 mg/l, respectively. On the other hand, Cd^2+^ toxicity was alleviated in the presence of TiO_2_-ENs. Algal growth was less suppressed in treatments with comparable total Cd^2+^ concentration but more TiO_2_-ENs. However, such toxicity difference disappeared and all the data points could be fitted to a single Logistic dose-response curve when cell growth inhibition was plotted against the free Cd^2+^ concentration. No detectable amount of TiO_2_-ENs was found to be associated with the algal cells. Therefore, TiO_2_-ENs could reduce the free Cd^2+^ concentration in the toxicity media, which further lowered its bioavailability and toxicity to *C. reinhardtii*.

## Introduction

Engineered nanoparticles (ENs), defined as man-made materials smaller than 100 nm in at least two dimensions, are widely recognized as having versatile applications in a variety of areas [1]. However, the novel properties ENs possess may not necessarily be benign. Their potentially adverse effects have been intensively investigated in recent years [2]–[4]. The toxicity of ENs was found to be determined by several physicochemical parameters like particle size, shape, aggregation status, surface coating, chemical composition and so on [3]. Although examining the toxicity of ENs alone could give us invaluable information about the environmental and health risks of nanomaterials, they are actually present in the real world together with other pollutants, which necessitates our understanding about the combined effects of ENs and other toxicants. Colloids are substances with the size range (1–1000 nm) much wider than that of ENs (1–100 nm). They have been reported to be able to facilitate the contaminant transport in the environment (so-called ‘Colloidal Pump’) [5], [6] and further influence their bioavailability in a colloid, pollutant, and organism species specific manner [7], [8]. However, it remains largely unknown how ENs may interact with other pollutants already existing in the environment and how these interactions may influence the behavior, fate, and toxicity of each other.

Up till now there is still limited research about the effects of ENs on the bioavailability of other pollutants with contradictory results reported. Park et al. [9] found no accumulation of 17α-ethinylestradiol associated with nC_60_ aggregates in the zebrafish *Danio rerio* through dietary exposure. In contrast, TiO_2_-ENs could enhance the toxicity of tributyltin to abalone embryos possibly as a result of tributyltin adsorption onto TiO_2_-ENs followed by internalization into the embryos [10]. Similarly, the toxicity of various metals like Cd^2+^, Cu^2+^, As (V) was found to increase in the presence of either TiO_2_-ENs or carbon nanotubes [11]–[14]. However, the synergistic toxicity of TiO_2_-ENs and As (V) on *Ceriodaphnia dubia* was either aggravated or eliminated as determined by EN to metal ratio [15]. Pollutant-specific effects were also observed for the influences of C_60_ aggregates on the toxicity of atrazine, methylparathion, pentachlorophenol, and phenanthrene [16].

To further explore how ENs may influence the bioavailability of other pollutants, we investigated Cd^2+^ adsorption kinetics and equilibrium isotherm on polyacrylate-coated TiO_2_-ENs. Its bioaccumulation and toxicity in the freshwater green alga *Chlamydomonas reinhardtii* with and without TiO_2_-ENs were compared. Potential accumulation (including surface adsorption and internalization) of TiO_2_-ENs in the algal cells was also examined. TiO_2_-ENs were chosen because of their wide applications in various products like sunscreens, cosmetics, paints, and surface coatings [10]. There were 50,400 tons of TiO_2_-ENs produced in 2010, representing 0.7% of the overall TiO_2_ market. Their production is projected to further increase to 201,500 tons by 2015 [17]. Meanwhile, TiO_2_-ENs are relatively inert with negligible dissolution and have no remarkable effects on *C. reinhardtii* based on the results of our preliminary experiment. This would simplify the later explanation of the toxicity results. In addition, TiO_2_-ENs were used in most researches on the interactions between ENs and other pollutants, which make the comparison of our study with the literature possible. As bare TiO_2_-ENs without any surface coating are easy to form aggregates in aqueous solution [14], a surface-coated substitute with similar photochemical properties was applied to ensure the effects we observed came from the nano-sized (<100 nm) dispersions. The overall objective was thus to reveal the underlying mechanisms how TiO_2_-ENs may affect the bioavailability and toxicity of Cd^2+^ and to answer the question whether Cd^2+^ toxicity in the presence of TiO_2_-ENs could still be predicted with the classical Free Ion Activity Model (FIAM), in which the metal toxicity is determined by its free ion concentration in the ambient environment [18].

## Materials and Methods

### Phytoplankton culture conditions and TiO_2_-EN characterization

The axenic culture of the Chlorophyta *Chlamydomonas reinhardtii* used was originally obtained from the Institute of Hydrobiology, Chinese Academy of Sciences, Wuhan. The algal cells were maintained in an artificial freshwater WC medium [19]. Its pH was kept at 7.5±0.1 by 5 mM 3-(N-morpholino) propanesulfonic acid (MOPS). The temperature was 25°C with a light illumination of 50 µmol photons/m^2^/s in a 12∶12 Light-Dark cycle.

TiO_2_-ENs (anatase) in powder form were purchased from Vivo Nano (Toronto, Canada). Their primary particle size was approximately 1–10 nm as reported by the manufacturer. They were coated with hydrophilic sodium polyacrylate (*ca.* 74% of the total EN weight) and thus could be well dispersed in water. The TiO_2_-EN suspension in the base adsorption or toxicity medium below was further examined through a transmission electron microscope (TEM, JEM-200CX from JEOL, Tokyo, Japan) to ensure their good dispersibility as described by Miao et al. [20]. Fast freeze-drying method was adopted to eliminate the EN aggregation during the sample preparation. A dynamic light scattering particle sizer (DLS, ZetaPALS from Brookhaven Instruments, NY, USA) was also applied to determine the hydrodynamic diameter of TiO_2_-ENs and their surface charge.

### Kinetics and equilibrium isotherm study of Cd^2+^ adsorption by TiO_2_-ENs

A modified WC medium (WC_m_) was used as the base solution of all adsorption experiments (Table S1). Ethylenediaminetetraacetic acid (EDTA, 11.7 µM) in the normal WC medium was excluded given that it is a strong metal binding ligand and could remarkably reduce Cd^2+^ adsorption. Accordingly, the trace metal nutrient concentrations were turned down to avoid their unnecessary precipitation (e.g., Fe^3+^) or toxicity (e.g., Cu^2+^). There were five TiO_2_-EN concentration treatments (1.0, 3.0, 10.0. 30.0, and 100.0 mg/l TiO_2_-ENs) in duplicate for the kinetics experiment. The total Cd^2+^ concentration was fixed at 1 mg/l. Each replicate had 20 ml adsorption medium in a 50 ml polypropylene centrifuge tube, which had been pre-equilibrated with solutions of the same composition as those in the following experiment to minimize the Cd^2+^ loss on the tube wall. The whole experiment lasted for 6 h with 7 time points (0, 0.25, 0.5, 0.75, 1, 2 and 6 h). At each time point, 0.2 ml aliquot from each replicate was filtered through a 10 kilo Dalton (kD) ultracentrifuge with pore size approximately 1 nm (PALL Nanosep series). Both the ultrafiltrate and what was retained on the membrane were then digested in 1 ml ultrapure concentrated HNO_3_ under 60°C for at least 4 d. They were further diluted with Milli-Q water (18.2 MΩ) to 7% w/v before the Cd^2+^ concentrations were determined by a Thermo M6 atomic absorption spectrophotometer equipped with a GF95Z graphite furnace system (Thermo Fisher Scientific Inc., Waltham, MA, USA). The total Cd^2+^ concentration in the adsorption media without ultrafiltration was also measured at the beginning and end of the experiment for mass balance calculation.

As for the equilibrium isotherm experiment, the variation of Cd^2+^ adsorption with its ambient concentration (nominal total Cd^2+^ concentration - 0.003, 0.01, 0.03, 0.1, 0.3, 1.0, 3.0, and 10.0 mg/l) was examined in the presence of 1, 10, and 100 mg/l TiO_2_-ENs, respectively. Since Cd^2+^ adsorption got saturated when its concentration approached 0.3 mg/l with 1 mg/l TiO_2_-ENs, the two highest Cd^2+^ concentrations (3.0 and 10.0 mg/l) were not used for this EN concentration treatment. The whole procedure was similar to that of the adsorption kinetics experiment above. Based on the results of the kinetics study, Cd^2+^ adsorption got equilibrated within 30 min. The duration of the equilibrium isotherm experiment was thus shortened to 4 h and Cd^2+^ distribution in different fractions was measured only at the end of this experiment. A control experiment with the same concentrations of Cd^2+^ but no TiO_2_-ENs was also conducted to examine the possibility of Cd^2+^ precipitation at different concentrations.

### Effects of TiO_2_-ENs on Cd^2+^ toxicity

Three toxicity tests in total were performed to investigate how TiO_2_-ENs may affect the bioavailability and toxicity of Cd^2+^ to *C. reinhardtii*. WC_m_ also served as the base of the toxicity media. There were seven Cd^2+^ concentration treatments (nominal total Cd^2+^ concentration - 0, 0.1, 0.3, 0.5, 0.8, 1.0, and 3.0 mg/l) in duplicate with and without 100 mg/l TiO_2_-ENs, respectively, for two of the three toxicity tests. However, the nominal total Cd^2+^ concentration was fixed at 1 mg/l and various concentrations (0, 1, 3, 10, 30, and 100 mg/l) of TiO_2_-ENs were applied in the third one. Major differences between the three toxicity tests were shown in Table S2. The polycarbonate bottles and other containers to be used in the toxicity tests were pre-equilibrated with the corresponding toxicity media similar to what was performed in the adsorption experiment above. All the toxicity media were made one day in advance and left overnight under the same conditions as the following experiment for equilibration. Their pH was kept at 7.5±0.1.

The algal cells were first acclimated in WC_m_ without Cd^2+^ or TiO_2_-ENs until arriving at the mid-exponential growth phase. They were then collected by centrifugation at 1700 RCF, rinsed twice with 15 ml fresh WC_m_ and resuspended into the toxicity media. Right before the addition of algal cells, 0.2 ml aliquot from each medium replicate was filtered through a 10 kD membrane. The total Cd^2+^ concentration in the ultrafiltrate (non-adsorbed Cd^2+^) was measured, based on which the free Cd^2+^ concentration ([Cd^2+^]_F_) of each toxicity medium was calculated using the MINEQL+ software package (Version 4.5 from Environmental Research Software, Hallowell, ME, USA) with updated thermodynamic constants and the influence of ionic strength calibrated. The whole experiment lasted for 2 d with three time points (0, 1^st^, and 2^nd^ d). At each time point, the cell density was measured by a Z2 Coulter Counter (Beckman Coulter Inc., CA, USA). The cell specific growth rate μ was calculated as described by Miao et al. [21]. At the end of each toxicity test, 10 ml aliquot from each replicate was filtered through a 1.2 µm polycarbonate membrane (Millipore). Cd^2+^ weakly adsorbed on the cell surface ([Cd^2+^]_cell-ads_) was removed after soaking the cells in 10 ml EDTA (100 µM) for 3 min. The algal cells retained on the membrane were further digested with concentrated HNO_3_ and [Cd^2+^]_intra_ was thus obtained. Meanwhile, the Cd^2+^ concentrations in the <1.2 µm filtrate, in the <10 kD fraction, and in the 1 ml aliquot without any filtration were measured for mass balance calculation.

To further examine the potential bioaccumulation of TiO_2_-ENs in the algal cells, another 10 ml aliquot was filtered through a 1.2 µm polycarbonate membrane, rinsed twice with 15 ml fresh WC_m_ and then combusted in muffle furnace at 460°C for 2 h. The residue was digested in a mixture of 0.4 g (NH_4_)_2_SO_4_ and 1.0 ml H_2_SO_4_ at 250°C for half an hour. After being diluted to 2.5% w/v by Milli-Q water, the Ti concentration was determined by GFAAS. Controls containing the same concentrations of TiO_2_-ENs and Cd^2+^ but no *C. reinhardtii* were applied to eliminate any interference from the TiO_2_-EN aggregates retained by the 1.2 µm polycarbonate membrane. The background Ti concentration in the cells not exposed to TiO_2_-ENs was also measured.

TEM images of algal cells exposed to 100 mg/l TiO_2_-ENs but without any addition of Cd^2+^ were then taken to visually examine the interactions between TiO_2_-ENs and *C. reinhardtii*. The sample preparation was similar to our previous study [20]. Briefly, 100 ml algal culture was centrifuged and fixed with 4% glutaraldehyde at 4°C for 4 h. After the cells were cleaned with phosphate buffer (0.3 M, pH = 7.3), they were stained in 1% (mg/ml, weight to volume ratio) osmium tetroxide for 2 h, and then dehydrated with 30%, 50%, 70%, 80%, 90% and 100% acetone solution sequentially. Afterwards, they were embedded into epoxy resin (Epon 812, DDSA, MNA and DMP-30), sectioned at 100 nm thickness, further stained with uranyl acetate (5 g in 50 ml ethanol) and lead citrate (1.33 g in 30 ml H_2_O). The elemental composition of the interesting spots on the TEM images was investigated with an energy dispersive X-ray (EDX) spectrometer.

### Statistical analysis

Any ‘significant’ difference (accepted at *p*<0.05) was based on results of one-way or two-way analysis of variance with post-hoc multiple comparisons (Turkey or Tamhane) (SPSS 11.0 by SPSS, Chicago, USA). The normality (Kolmogorov–Smirnov and Shapiro–Wilk tests) and homogeneity of variance (Levene's test) of the data were both examined when performing the analysis of variance.

## Results and Discussion

### Adsorption of Cd^2+^ by TiO_2_-ENs

**Figure 1:**
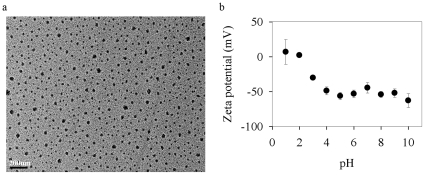
The transmission electron microscope image of TiO_2_-ENs dispersed in the modified WC medium (WC_m_) (a) and their zeta potentials (mV) at different pH (b).

The TiO_2_-ENs used in the present study were coated with sodium polyacrylate and could thus be well dispersed in WC_m_ as supported by their TEM images shown in Fig. 1a. Their diameter was 46.6 nm on average by measuring 1000 particles randomly chosen from the copper grids, which was consistent with what was obtained by DLS (19.0–46.8 nm). The relatively good dispersibility of TiO_2_-ENs coated by the polyelectrolyte could be explained by their much lower pH_pzc_ (Fig. 1b), at which a particle surface has zero net electrical charge, than that of their naked counterpart (pH_pzc_ = 2 vs. 6) [22], [23]. Such decrease in pH_pzc_ was mainly caused by polyacrylate's ability to push the slip plane of the crystal lattice away from the ENs, change their charge distribution in the diffusion layer and block the active sites on the TiO_2_-EN surface as well [23]. Furthermore, the extent of the shift in pH_pzc_ was determined by both the concentration and molecular weight of polyacrylates. Despite their good dispersibility in WC_m_, the actual diameter of TiO_2_-ENs was much bigger than what was reported by the manufacturer (1–10 nm) suggesting the electric double layer of the primary nanoparticles was compressed in the adsorption medium with the ionic strength 2.65×10^−3^ M and aggregates were thus formed. The presence of divalent cations like Ca^2+^ (0.25 mM) and Mg^2+^ (0.15 mM) in WC_m_ could further destabilize the TiO_2_-EN suspension [1].

**Figure 2:**
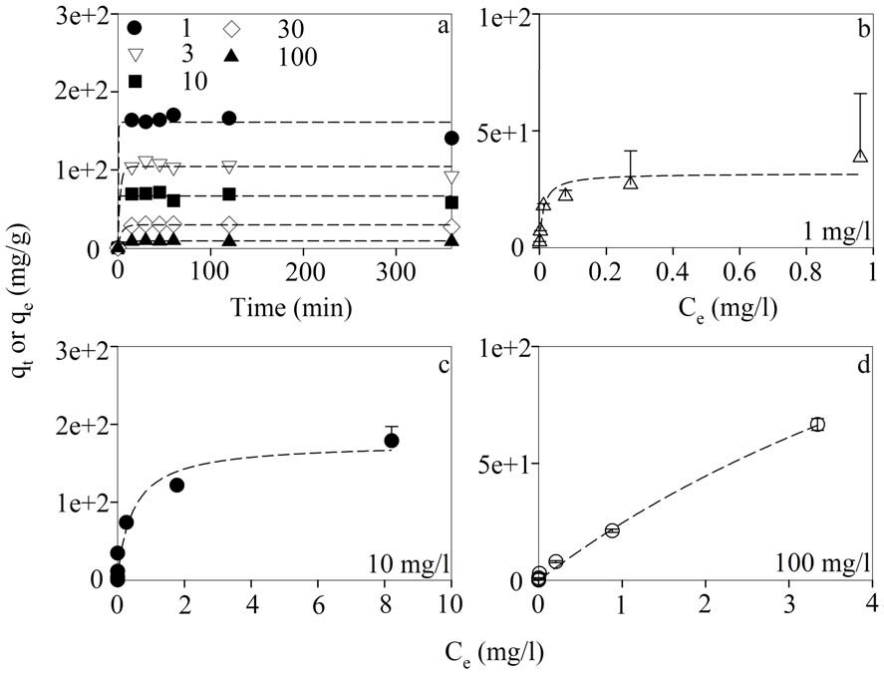
Adsorption of Cd^2+^ (q_t_, mg/g) on TiO_2_-ENs in the kinetics (a) and 4-h equilibrium isotherm (b–d) experiments, respectively. There were five treatments with different concentrations of TiO_2_-ENs (1.0, 3.0, 10.0. 30.0, and 100.0 mg/l) but the same concentration of total Cd^2+^ (1 mg/l) in the kinetics experiment. Various concentrations of Cd^2+^ (0.003, 0.01, 0.03, 0.1, 0.3, 1.0, 3.0, and 10.0 mg/l initially) were used in each equilibrium isotherm experiment with different TiO_2_-EN concentrations (b–d: 1, 10, 100 mg/l, respectively). Since Cd^2+^ adsorption got saturated when its concentration approached 0.3 mg/l with 1 mg/l TiO_2_-ENs, the two highest Cd^2+^ concentrations (3.0 and 10.0 mg/l) were not used for this EN concentration treatment. Dashed lines represent the simulated curves of Cd^2+^ adsorption kinetics and equilibrium isotherm by the pseudo-first order (a) and Langmuir (b–d) models. Data are mean ± standard deviation (n = 2).

In both adsorption kinetics and equilibrium isotherm experiments, the concentrations of Cd^2+^ retained on the 10 kD membrane (TiO_2_-EN surface-adsorbed) and in the filtrate (non-adsorbed) were compared with what was measured without filtration. A good mass balance (100±10%) was achieved for most treatments. Cd^2+^ was found to quickly adsorb onto TiO_2_-ENs and a steady state was reached within 30 min (Fig. 2a). Rapid association of Cd^2+^, Cu^2+^, Ni^2+^, Pb^2+^ and Zn^2+^ with TiO_2_-ENs was also observed by Engates and Shipley [24] and most adsorption was completed in 5 min. Meanwhile, higher proportions of Cd^2+^ were adsorbed in higher TiO_2_-EN concentration treatments unless no Cd^2+^ in the medium was available any more when the concentration of TiO_2_-ENs exceeded 30 mg/l. Accordingly, 0.16, 0.33, 0.70, 0.90, and 0.93 mg/l Cd^2+^ was adsorbed by 1, 3, 10, 30, 100 mg/l TiO_2_-ENs, respectively, after 30 min. Such trend looked reversed in Fig. 2a as the adsorption was normalized to the TiO_2_-EN concentration (mg/g). Additionally, the possibility whether Cd^2+^ with concentration up to 1 mg/l was over-saturated in WC_m_, precipitated out and thus made the Cd^2+^ adsorption results overestimated was also investigated. A negligible amount (less than 1%) was retained on the 10 kD membrane without any addition of TiO_2_-ENs at the end of the 6-h experiment suggesting that all the Cd^2+^ was in soluble form for the kinetics experiment. Although EDTA was not used in WC_m_, no significant precipitates were formed when preparing this medium.

The Cd^2+^ adsorption kinetics results were then fitted with the pseudo-first order equation as follows,
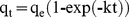
(1)Where q_t_ and q_e_ are the Cd^2+^ adsorption (mg/g) at time t (min) and at equilibrium, respectively. k (min^−1^) represents the equilibrium rate constant of the pseudo-first order adsorption. Values of the different parameters thus obtained were listed in Table 1. The adsorption of Cd^2+^ onto TiO_2_-ENs and Cu^2+^ onto Fe_3_O_4_ magnetic ENs were also found to comply with the pseudo-first order model in previous studies [22], [25]. Values of k were 0.15–0.25 min^−1^ for Cd^2+^ and 0.64–1.05 min^−1^ for Cu^2+^, which were of the same order of magnitude as what was found (0.23–2.35 min^−1^) in the present study even though the TiO_2_-ENs used here were coated with sodium polyacrylate. Fitting the adsorption kinetics data points by the pseudo-second order model was also tried with unsatisfied results (data not shown), possibly due to the high Cd^2+^ concentrations we used considering that the choice of models is dependent on the solute concentration [26].

**Table 1:** Values of the different parameters obtained when simulating the kinetics and equilibrium isotherm of Cd^2+^ adsorption by TiO_2_-ENs with the pseudo-first order and Langmuir models, respectively.

Pseudo-first order				Langmuir			
q_e_	k	*r^2^*	*p*	q_m_	K_a_	*r^2^*	*p*
160.9±12.1	2.35±0.35	0.98	<0.0001	31.9±17.2	83.5±49.6	0.88	0.0053
104.1±2.90	0.39±0.05	0.98	<0.0001	177.1±77.4	2.14±1.12	0.94	<0.0001
66.1±1.32	3.21±0.48	0.96	<0.0001	242.2±23.9	0.113±0.04	0.99	<0.0001
29.2±0.63	0.25±0.01	0.99	<0.0001				
8.51±0.30	0.23±0.03	0.97	<0.0001				

In the equilibrium isotherm experiment, a biphasic correlation between the TiO_2_-EN normalized Cd^2+^ adsorption (q_e_, mg/g) and the non-adsorbed Cd^2+^ concentration in the medium (C_e_, mg/l) was found for each of the three TiO_2_-EN concentration treatments (1, 10, and 100 mg/l) (Fig. 2b–d). Namely, q_e_ went up proportionally with C_e_ first, slowed down thereafter and even plateaued at high Cd^2+^ levels especially when 1 or 10 mg/l TiO_2_-ENs were used. In the meantime, the potential difference in the proportions of Cd^2+^ adsorbed by different concentrations of TiO_2_-ENs was small or negligible when the initial concentration of Cd^2+^ was too low to saturate the ENs. However, the difference got more and more significant as Cd^2+^ adsorption approached the saturation point. When 3 µg/l Cd^2+^ was applied, most of it (91.7–97.3%) was adsorbed in all the three TiO_2_-EN concentration treatments. As the initial Cd^2+^ concentration increased further to 0.01 and 1 mg/l, the proportion of Cd^2+^ complexed with 1 mg/l TiO_2_-ENs decreased to 71.8% and 9.02%. However, nearly all Cd^2+^ (96.4–100%) could still be adsorbed on 10 and 100 mg/l TiO_2_-ENs. It was not until the initial Cd^2+^ concentration exceeded 1 mg/l that significant difference (*p*<0.05) between the 10 and 100 mg/l TiO_2_-EN concentration treatments appeared with 17.9% and 66.6% adsorption when the initial Cd^2+^ concentration was 10 mg/l. The concentration of Cd^2+^ retained on the 10 kD membrane in the control treatments having the same concentrations of Cd^2+^ but no TiO_2_-ENs was also negligible as compared with those adsorbed by the ENs. It further implies that Cd^2+^ precipitation was insignificant for all the concentration treatments of the present study as was in contrast to what was estimated by MINEQL+ based on an equilibrium assumption.

The biphasic correlation between q_e_ and C_e_ was then fitted to the Langmuir isotherm for each of the three TiO_2_-EN concentration treatments as follows,
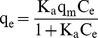
(2)Where K_a_ is the Langmuir constant (l/mg) related to adsorption energy and q_m_ represents the maximal monolayer adsorption capacity (mg/g). A good correlation was found with values for the various parameters shown in Table 1 suggesting a monolayer adsorption of Cd^2+^ on TiO_2_-ENs.

Cd^2+^ adsorption on TiO_2_-ENs with different crystal size (7.72–145 nm) was previously investigated [27]. Values of q_m_ thus derived from the Langmuir isotherm were in the range of 3.93–56.0 mg/g with lower adsorption by bigger particles. Much smaller difference was observed when q_m_ was normalized to the surface area of TiO_2_-ENs (0.29–0.39 mg/m^2^). As the TiO_2_-ENs we used were made up of a TiO_2_ core (4.23 g/cm^3^) coated with sodium polyacrylate (1.22 g/cm^3^), its density and specific surface area were estimated to be approximately 1.50 g/cm^3^ and 85.8 m^2^/g. However, the surface area normalized q_m_ (0.37–2.82 mg/m^2^) we obtained was still higher than what was reported by Gao et al. [27], especially for the two higher TiO_2_-EN concentration treatments. It suggests that the polyacrylate surface coating could improve the metal ion adsorption ability of the ENs. Cd^2+^ was able to form bidentate and monodentate ligand complex with polyacrylic acid [28], which may also be the same case for its adsorption on the polyacrylate-coated TiO_2_-ENs. Given that sodium polyacrylate accounts for 74% of the TiO_2_-ENs we used, the Cd^2+^ to –COOH ratio at saturation would be 0.036, 0.20, and 0.27, respectively, when the concentration of TiO_2_-ENs was 1, 10, and 100 mg/l. It implies that part of the carboxylate group from polyacrylate was bound with TiO_2_ or other cations (e.g., Ca^2+^ and Mg^2+^ etc.) in the adsorption medium and was thus not available to Cd^2+^. The possibility that the EN surface was heterogeneous and some of the Cd^2+^ may be complexed with the TiO_2_ core itself further complicated the metal-EN interactions [27].

### Cd^2+^ toxicity as affected by TiO_2_-ENs

**Figure 3:**
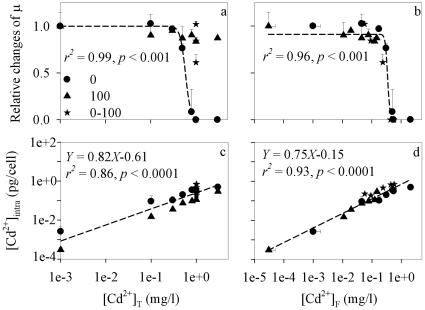
Relative changes of the cell specific growth rate (μ) (a–b) and intracellular Cd^2+^ concentration ([Cd^2+^]_intra_, pg/cell) (c–d) with either the total dissolved ([Cd^2+^]_T_, mg/l) (a, c) or free Cd^2+^ ([Cd^2+^]_F_, mg/l) concentrations (b, d) at the beginning of the three toxicity experiments where 0, 100, and 1–100 mg/l TiO_2_-ENs were applied, respectively. Dashed lines represent the simulated curves for the relative changes of μ (a–b) and [Cd^2+^]_intra_ (c–d) at different [Cd^2+^]_T_ (a, c) and [Cd^2+^]_F_ (b, d) by the Logistic dose-response and Freundlich models, respectively. Data are mean ± standard deviation (n = 2).

The growth of *C. reinhardtii* in WC_m_ (no addition of Cd^2+^) with or without 100 mg/l TiO_2_-ENs was compared in our preliminary experiment. No obvious growth inhibition was found, which simplified our exploration how TiO_2_-ENs may affect the bioavailability and toxicity of metal ions. The toxicity of bare TiO_2_-ENs to various phytoplankton was investigated in the literature [29]. The median effect concentration EC50 thus obtained ranged over a few orders of magnitude (e.g., 5.83–241 mg/l) as the toxicity of ENs was dependent on parameters like particle size, shape, chemical composition and so on. Surface coatings may further eliminate the toxicity of ENs [30]. The initial and final Cd^2+^ concentrations in the <10 kD fraction of each replicate for the three toxicity tests were both measured. The decrease in [Cd^2+^]_F_ with exposure time was within 30% for most treatments. The relative changes of μ were thus plotted against either total dissolved Cd^2+^ concentration ([Cd^2+^]_T_, including what was adsorbed on TiO_2_-ENs) or [Cd^2+^]_F_ (Fig. 3a, b) at the beginning of each toxicity test to determine which metal ion concentration could predict its toxicity better in the presence of TiO_2_-ENs. As expected, μ was substantially reduced at high Cd^2+^ levels when no TiO_2_-ENs was applied. A typical dose-response correlation between the relative changes of μ and either type of Cd^2+^ concentration was observed. Namely, the cell growth was kept constant in the first three lowest Cd^2+^ concentration treatments. It was then strikingly inhibited with more than 90% reduction when [Cd^2+^]_T_ ([Cd^2+^]_F_) increased to 0.8 (0.50) mg/l and completely ceased thereafter.

However, when 100 mg/l TiO_2_-ENs were applied to the different treatments with [Cd^2+^]_T_ comparable to those of the first toxicity test above, the adverse effects of Cd^2+^ were substantially alleviated. There was a growth inhibition of only 13% in the highest concentration treatment with [Cd^2+^]_T_ 3 mg/l, which in contrast was lethal to the cells if no TiO_2_-ENs were added. Similarly, μ went down from 0.46 d^−1^, as was comparable to that in the control without any addition of TiO_2_-ENs and Cd^2+^, to zero when [Cd^2+^]_T_ was fixed at 1 mg/l but the TiO_2_-EN concentration decreased from 100 to 0 mg/l in the third experiment. However, growth inhibition to different extent at similar [Cd^2+^]_T_ but different concentrations of TiO_2_-ENs, as shown in Fig. 3a, disappeared when the relative changes of μ were plotted against [Cd^2+^]_F_. All the data points from the different toxicity tests could be well fitted to a single Logistic dose-response curve (y = min+(max−min)/(1+(x/EC50)^Hillslope^)) (Fig. 3b). The [Cd^2+^]_F_-based EC50 thus obtained was 0.35±0.03 mg/l which was similar to 0.46 mg/l observed in our previous study [31] and was within the range of values (0.03–2.41 mg/l) reported in the literature [32], [33]. As the cell growth was differently inhibited at similar [Cd^2+^]_T_ but various concentrations of TiO_2_-ENs, only the dose-related responses of the first toxicity test without any addition of TiO_2_-ENs were simulated with the Logistic model in Fig. 3a.

Of the limited research on the interactions between ENs and trace metals, TiO_2_-ENs were frequently chosen which made the comparison of our study with the literature possible. The bioaccumulation of Cd^2+^ and As (V) in carp was found to increase remarkably in the presence of TiO_2_-ENs, as was explained by ENs' ability to facilitate the metal transport through the gills (dissolved uptake) and to induce the metal assimilation in the intestines (dietary assimilation) when EN-contaminated foods were fed to the fish [34], [35]. Additionally, the enhanced oxidation of metal ions in reduced form like As (III) by TiO_2_-ENs as a photocatalyst especially under the light condition could further accelerate the metal uptake [34]. As a result, the trace metal toxicity was aggravated even though part of them were still associated with TiO_2_-ENs in the organisms [12]. On the other hand, metal toxicity to unicellular organisms may decline abruptly in the presence of TiO_2_-ENs if the metal-EN complexes thus formed cannot penetrate the cell membrane. The bare TiO_2_-ENs used by Hartmann et al. [14] were able to reduce the Cd^2+^ toxicity to a green alga *Pseudokirchneriella subcapitata* but the toxicity inhibition was greater than what could be explained by the concentration of Cd^2+^ not associated with TiO_2_-ENs, suggesting a possible carrier effect, or mixture toxic effects of TiO_2_-ENs and Cd^2+^. The latter possibility was more likely as considering the toxicity of TiO_2_-ENs on the alga itself (EC50 = 71.1–241 mg/l) and any direct evidence of EN internalization was lacking.

To further examine the underlying mechanisms how Cd^2+^ toxicity was lessened by the polyacrylate-coated TiO_2_-ENs, the bioaccumulation of Cd^2+^ ([Cd^2+^]_cell-ads_ and [Cd^2+^]_intra_) was quantified at the end of each toxicity test. Their changes with [Cd^2+^]_T_ as well as [Cd^2+^]_F_ were shown in Fig. 3c, d and S1. Overall, there was a positive correlation between Cd^2+^ accumulation and [Cd^2+^]_T_ or [Cd^2+^]_F_. When [Cd^2+^]_F_ went up from 3.05×10^−5^ to 2.10 mg/l, [Cd^2+^]_intra_ was enhanced by three orders of magnitude (Fig. 3d). The cellular Cd^2+^ concentration in the same strain of alga was found to increase approximately from 0.025 pg/cell when [Cd^2+^]_F_ was 5.39×10^−3^ mg/l to 0.25 pg/cell with [Cd^2+^]_F_ 3.37 mg/l [33], as was comparable to what was observed in the present study. On the other hand, [Cd^2+^]_intra_ was strikingly different in treatments containing the same [Cd^2+^]_T_ but various concentrations of TiO_2_-ENs. It was decreased by 40–88% in all treatments when 100 mg/l TiO_2_-ENs were applied in the second toxicity test as compared with those in the first one (Fig. 3c). Similarly, a negative correlation between [Cd^2+^]_intra_ and TiO_2_-EN concentration was observed with fixed [Cd^2+^]_T_ in the third toxicity experiment.

More importantly, the difference between [Cd^2+^]_intra_ at similar [Cd^2+^]_T_ but distinct concentrations of TiO_2_-ENs was substantially diminished when [Cd^2+^]_intra_ was plotted against [Cd^2+^]_F_ instead of [Cd^2+^]_T_ (Fig. 3d). Such trend was more obvious when all the data points from the three toxicity tests were fitted to a single Freundlich isotherm below for each diagram (Fig. 3c, d and S1).
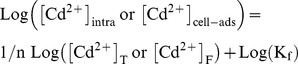
(3)Where K_f_ represents the Freundlich constant and n is a dimensionless parameter related to the metal binding affinity. A better correlation between [Cd^2+^]_intra_ and [Cd^2+^]_F_ than that between [Cd^2+^]_intra_ and [Cd^2+^]_T_ (*r^2^* = 0.93 vs. 0.86) was observed. It suggests that Cd^2+^ toxicity alleviation by TiO_2_-ENs was mainly caused by the decrease in [Cd^2+^]_F_ as a result of surface adsorption. In contrast, there was an unsatisfied correlation (*r^2^* = 0.68 vs. 0.62) between [Cd^2+^]_cell-ads_ and [Cd^2+^]_T_ or [Cd^2+^]_F_ (Fig. S1). At similar [Cd^2+^]_F_, higher Cd^2+^ adsorption was usually found when TiO_2_-ENs were applied. Therefore, a certain amount of TiO_2_-ENs might be associated with the algal cells or just highly aggregated in the medium and were retained by the 1.2 µm polycarbonate membrane. Cd^2+^ adsorbed on these ENs could also be removed by EDTA, and thus made [Cd^2+^]_cell-ads_ overestimated.

**Figure 4:**
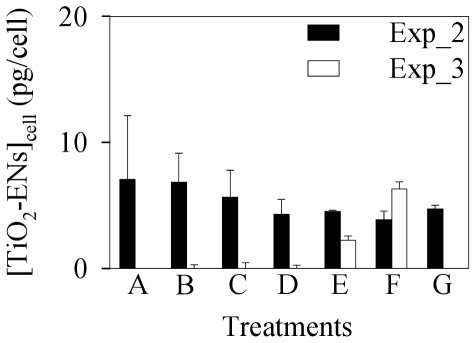
Accumulation of TiO_2_-ENs ([TiO_2_-ENs]_cell_, pg/cell) by *Chlamydomonas reinhardtii* in the different treatments of the second (Exp_2) and third (Exp_3) toxicity experiments. In Exp_2, treatment A–G indicates different initial concentrations of Cd^2+^ (0, 0.1, 0.3, 0.5, 0.8, 1.0, and 3.0 mg/l) with the TiO_2_-EN concentration fixed at 100 mg/l. In Exp_3, the initial Cd^2+^ concentration was fixed at 1 mg/l and various concentrations (0, 1, 3, 10, 30, and 100 mg/l) TiO_2_-ENs were used for treatments A–F. Data are mean ± standard deviation (n = 2).

**Figure 5:**
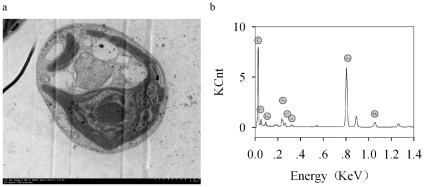
A representative transmission electron microscope (TEM) image (a) and the elemental composition of the interesting spots on it (b), as investigated with an energy dispersive X-ray (EDX) spectrometer, for a cell slice of *C. reinhardtii* exposed to 100 mg/l TiO_2_-ENs but without any addition of Cd^2+^.

Potential TiO_2_-EN accumulation (including both cell surface adsorbed and intracellular accumulated ones) by *C. reinhardtii* was then quantified with GFAAS. As shown in Fig. 4, the bioaccumulated concentration of TiO_2_-ENs ([TiO_2_-ENs]_cell_) was in the range of 3.85–7.06 pg/cell for the second toxicity experiment in the presence of 100 mg/l TiO_2_-ENs. Although [TiO_2_-ENs]_cell_ decreased slightly with the enhancement of [Cd^2+^]_T_, it was statistically insignificant (*p*>0.05). Additionally, a substantial accumulation of TiO_2_-ENs was only detected in the two highest TiO_2_-EN concentration treatments (30 and 100 mg/l) for the third toxicity test. Given that TiO_2_-ENs had a diameter of 46.6 nm on average, there should be 4.8×10^4^–8.9×10^4^ particles associated with a single algal cell as equivalent to approximately 1000 particles within each cell slice (100 nm thick) mounted on the TEM copper grid when 100 mg/l TiO_2_-ENs were applied. However, no TiO_2_-ENs were found either inside the cells or adsorbed on the cell surface after several cell slices were investigated with suspicious spots scanned by the EDX spectrometer (Fig. 5). It implies that most of the Ti signal determined by GFAAS might come from the additional TiO_2_-EN aggregates intercepted by the 1.2 µm membrane in the presence of *C. reinhardtii*, which cannot be subtracted with the control treatments containing the same concentrations of Cd^2+^ and TiO_2_-ENs but no algal cells. TiO_2_-ENs can attach to various algal species. The green alga *P. subcapitata* could even carry TiO_2_-ENs with weight 2.3 times higher than their own on the cell surface [14], [36], [37]. The adsorption of TiO_2_-ENs was found to be dependent on the pH of the medium and maximum adsorption was observed at pH = 5.5 (comparable to the pH_pzc_ of bare TiO_2_-ENs) [38]. It implies that electrostatic attraction played a critical role in the interactions between TiO_2_-ENs and algal cells. As the pH_pzc_ of TiO_2_-ENs we used is around 2, their surface was negatively charged in WC_m_ (pH = 7.5) the same as that of the algal cells themselves. Therefore, a negligible amount of TiO_2_-ENs would be expected to be associated with *C. reinhardtii* unless other forces such as hydrogen bonding overrides the electrostatic and steric repulsion between the cells and ENs as observed by Schwab et al. [39]. The lack of direct contact between polyacrylate-coated TiO_2_-ENs might be another reason why they were less toxic than bare TiO_2_-ENs.

Overall, the TiO_2_-ENs used in the present study could adsorb Cd^2+^ rather quickly with the maximum adsorption capacity ranging from 31.9 to 242.2 mg/g. The electrostatic and potentially steric repulsions between TiO_2_-ENs and algal cells could hinder their direct contact with each other, thus prevent the internalization of TiO_2_-ENs into the cells. The toxicity of Cd^2+^ was alleviated considerably when TiO_2_-ENs were applied, as Cd^2+^ adsorption on the ENs decreased its free ion concentration in the toxicity medium and further its bioaccumulation in the algal cells. However, the Cd^2+^ toxicity in the presence of TiO_2_-ENs could still be well predicted with the classical FIAM model.

## Supporting Information

Figure S1 **Relative changes of the cell surface adsorbed Cd^2+^ concentration ([Cd^2+^]_cell-ads_, pg/cell) with either the total dissolved ([Cd^2+^]_T_, mg/l) (a) or free Cd^2+^ ([Cd^2+^]_F_, mg/l) concentrations (b) at the beginning of the three toxicity experiments where 0, 100, and 1–100 mg/l TiO_2_-ENs were applied, respectively.** Dashed lines represent the simulated curves of [Cd^2+^]_cell-ads_ at different [Cd^2+^]_T_ (a) and [Cd^2+^]_F_ (b) by the Freundlich isotherm model. Data are mean ± standard deviation (n = 2).
(TIF)

Table S1 **Compounds and their concentrations in the modified WC medium used in the present study.**
(DOC)

Table S2 **Composition of the toxicity media for the three experiments.**
(DOC)
