# Supplementary figures and images for: Cd2+ Toxicity to a Green Alga Chlamydomonas reinhardtii as Influenced by Its Adsorption on TiO2 Engineered Nanoparticles
Source: PLoS One. 2012 Mar 5;7(3):e32300. doi: 10.1371/journal.pone.0032300 (PMC3293805; doi:10.1371/journal.pone.0032300)

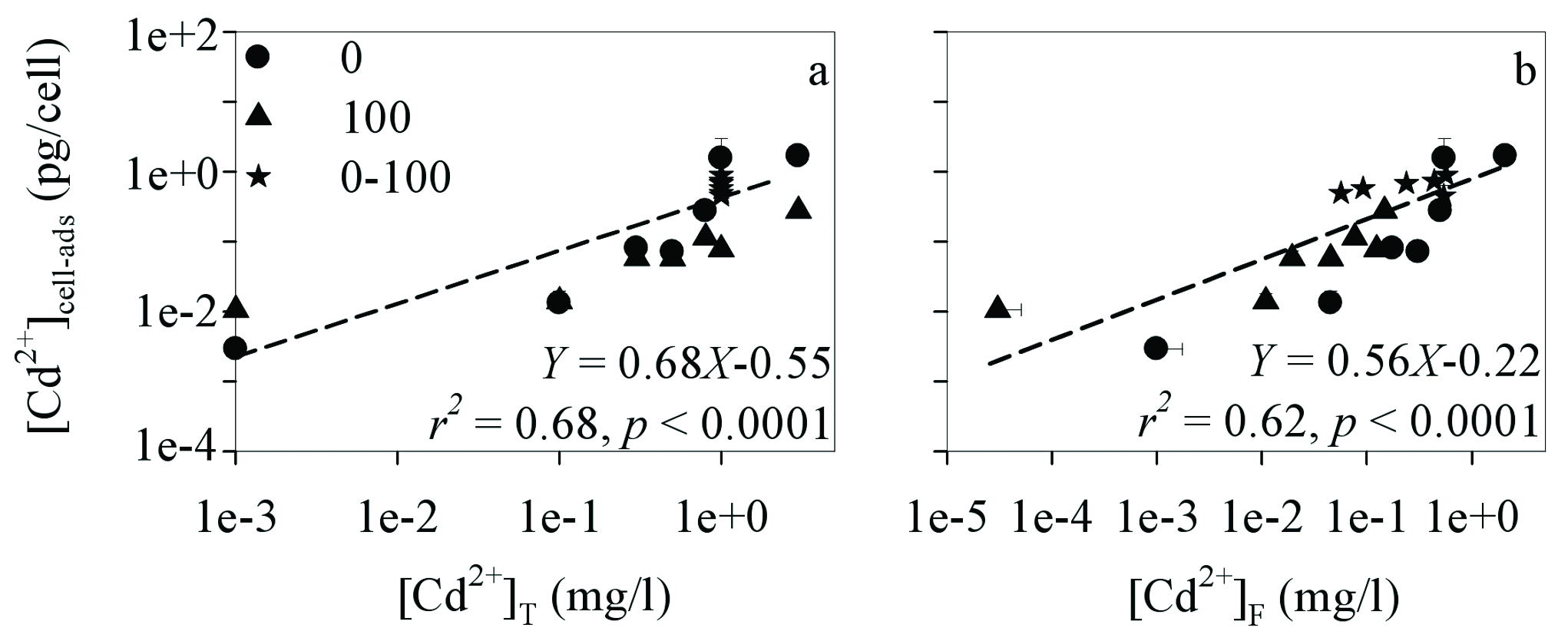

Supplement: Figure S1 — Relative changes of the cell surface adsorbed Cd2+ concentration ([Cd2+]cell-ads, pg/cell) with either the total dissolved ([Cd2+]T, mg/l) (a) or free Cd2+ ([Cd2+]F, mg/l) concentrations (b) at the beginning of the three toxicity experiments where 0, 100, and 1–100 mg/l TiO2-ENs were applied, respectively. Dashed lines represent the simulated curves of [Cd2+]cell-ads at different [Cd2+]T (a) and [Cd2+]F (b) by the Freundlich isotherm model. Data are mean ± standard deviation (n = 2). (TIF) [file pone.0032300.s001.tif]
