# Supplementary material for: Cd2+ Toxicity to a Green Alga Chlamydomonas reinhardtii as Influenced by Its Adsorption on TiO2 Engineered Nanoparticles
Source: PLoS One. 2012 Mar 5;7(3):e32300. doi: 10.1371/journal.pone.0032300 (PMC3293805; doi:10.1371/journal.pone.0032300)
Supplement: Table S1 — Compounds and their concentrations in the modified WC medium used in the present study. (DOC) [file pone.0032300.s002.doc]

Table S1. Compounds and their concentrations in the modified WC medium used in the present study.

| Component | Final Concentration | Component | Final Concentration |
| --- | --- | --- | --- |
| NaNO_3_ | 1000 μM | MnCl_2_ | 0.1 μM |
| K_2_HPO_4_ | 50 μM | ZnSO_4_ | 10 nM |
| MgSO_4_ | 150 μM | Na_2_MoO_4_ | 10 nM |
| CaCl_2_ | 250 μM | CuSO_4_ | 0.05 nM |
| NaHCO_3_ | 150 μM | CoCl_2_ | 5 nM |
| Na_2_SiO_3_ | 100 μM | MOPS | 5 mM |
| FeCl_3_ | 0.5 μM |  |  |
