# Supplementary material for: Cd2+ Toxicity to a Green Alga Chlamydomonas reinhardtii as Influenced by Its Adsorption on TiO2 Engineered Nanoparticles
Source: PLoS One. 2012 Mar 5;7(3):e32300. doi: 10.1371/journal.pone.0032300 (PMC3293805; doi:10.1371/journal.pone.0032300)
Supplement: Table S2 — Composition of the toxicity media for the three experiments. (DOC) [file pone.0032300.s003.doc]

Table S2. Composition of the toxicity media for the three experiments.

| Experiment | Composition |
| --- | --- |
| 1^st^ toxicity test | WC_m_ + 0, 0.1, 0.3, 0.5, 0.8, 1.0, or 3.0 mg/l Cd^2+^ |
| 2^nd^ toxicity test | WC_m_ + 0, 0.1, 0.3, 0.5, 0.8, 1.0, or 3.0 mg/l Cd^2+^ + 100 mg/l TiO_2_-ENs |
| 3^rd^ toxicity test | WC_m_ + 1.0 mg/l Cd^2+^ + 0, 1, 3, 10, 30, or 100 mg/l TiO_2_-ENs |
